# Pharmaceutical Advantages of GenoTX-407, A Combination of Extracts from *Scutellaria baicalensis* Root and *Magnolia officinalis* Bark

**DOI:** 10.3390/antiox9111111

**Published:** 2020-11-11

**Authors:** Eun-Jung Yoon, Mi Young Lee, Byoung Il Choi, Kyong Jin Lim, Seung Young Hong, Dongsun Park

**Affiliations:** 1 Department of Biology Education, Korea National University of Education, Cheongju 28173, Korea; 315836@naver.com; 2 Genogen Co., Ltd., Cheongju 28161, Korea; young2472@hanmail.net (M.Y.L.); fearon@hanmail.net (B.I.C.); dati98@naver.com (K.J.L.); aseung02a@naver.com (S.Y.H.)

**Keywords:** *Scutellaria baicalensis*, *Magnolia officinalis*, antioxidant activity, antimicrobial activity, anti-inflammatory activity

## Abstract

Background: Extracts of *Scutellaria baicalensis* root (SBR) and *Magnolia officinalis* barks (MOB) possess significant antioxidant, anti-inflammatory, and antimicrobial properties; however, these also exert adverse effects such as cytotoxicity. To overcome the adverse effects, we formulated a combination of the extracts, named GenoTX-407, with SBR and MOB extracts mixed in 5:1 ratio. The antioxidant, antimicrobial, and anti-inflammatory activities of SBR and MOB extracts and GenoTX-407 were evaluated. Methods: To optimize the extraction conditions of SBR and MOB, different ethanol concentrations and extraction times and treatments of the extracts with different solvents for varying time periods were tested. Anti-inflammatory activity was assessed via NO scavenging assay and analysis of anti-inflammatory activity-related gene expression in RAW 264.7 cells. Agar disk diffusion and microdilution assays were used to determine the antimicrobial activity. Antioxidant activity was evaluated through DPPH assay and analyses of peroxidation and antioxidant-related protein expression in HeLa cells. Results: Extraction with 0% ethanol for 2 h and 1.5% phosphoric acid for 0.5 h yielded maximum SBR extracts. For MOB, 50% ethanol extraction for 2 h followed by further extraction in hexane for 0.5 h yielded the highest extracts. SBR (46.1 ± 0.9 %) and MOB (48.9 ± 1.0 %) extracts effectively inhibited NO production, and dose-dependently reduced the expression of *TNF-α*, *iNOS*, *NF-κB*, *COX2*, and *IL-6*. MOB and GenoTX-407 inhibited the growth of *Escherichia coli*, *Staphylococcus aureus*, *Candida albicans*, and *Propionibacterium acnes*, as evidenced in disk diffusion and microdilution assays. SBR (EC50, 107.7 µg/mL and 38.3 µg/mL), MOB (62.41 µg/mL and 72.45 µg/mL), and GenoTX-407 (7.7 µg/mL and 26.4 µg/mL) exhibited excellent antioxidant potency and could scavenge free radicals of DPPH and lipid peroxidation; additionally, SOD, CAT, HO-1, and Nrf2 expression was increased in HeLa cells. SBR showed more potent antioxidant activity than MOB. Contrastingly, MOB exhibited more potent anti-inflammatory and antimicrobial activities than SBR. Interestingly, GenoTX-407 was the most efficient in all the assays, compared with SBR and MOB. Conclusion: This study demonstrated that GenoTX-407, the combination of SBR and MOB, is a potential drug candidate exerting antioxidant and anti-inflammatory effects via the Nrf2/HO-1 and NF-κB signaling pathways.

## 1. Introduction

Plants are a rich source of candidate compounds for drug development [1]. Previous studies have identified a plethora of plants with beneficial health effects. In recent decades, medicinal plants have garnered attention as a valuable source of therapeutic agents, and many of the presently used therapeutic drugs are plant-derived natural products or derivatives thereof [2].

Phenolic compounds are the most abundant antioxidants in medicinal plants and have been shown to play important roles in the prevention of diseases [3]. Polyphenol compounds, such as pyrogallol, abrogate kainic acid (KA)-induced neuronal damage through oxidative stress inhibition. Chlorogenic acid prevents gastritis through its anti-inflammatory activity [4]. Moreover, a variety of plant-derived phenolic compounds have been reported to possess distinct antioxidant and anti-inflammatory activities that contribute to their therapeutic functions [5].

*Scutellaria baicalensis*, a flowering plant species of the *Lamiaceae* family, is a rich source of such phenolic compounds [6,7]. A variety of flavonoids, including baicalin, have been isolated and characterized from *S. baicalensis*. Baicalin, which is a major bioactive compound [7], is used in traditional Chinese medicine for treating diarrhea, dysentery, hypertension, hemorrhage, insomnia, inflammation, and respiratory infections [8]. In particular, it exerts a potent antioxidant effect through the Nrf2/HO-1 pathway [9]. Magnolia trees are mainly distributed in East and Southeast Asia. The bark of *Magnolia officinalis*, which is used in traditional Chinese and Japanese medicines for the treatment of anxiety, asthma, depression, gastrointestinal disorders, headaches, and more [10,11], has been recently reported to possess pharmacological activities such as antioxidant, anti-inflammatory, antibiotic, and antispasmodic effects [12]. A previous study identified magnolol and honokiol as the major bioactive compounds in *M. officinalis* bark, and these showed potent anti-inflammatory effect through inhibition of the nuclear factor (NF)-κB signaling pathway [13].

Despite remarkable therapeutic effects, these plants also have several adverse effects including cytotoxicity, genotoxicity, carcinogenicity, and developmental toxicity [14,15,16]. To overcome these problems, many researchers have studied the effects of complex mixtures containing extracts from these plants in different combinations. Combinations of these extracts often showed positive synergistic effects that surpassed the activities of the individual components [17,18]. Therefore, in this study, we tried to optimize the extraction conditions for enhancing the phenolic compounds content in extracts of *S. baicalensis* roots (SBR) and *M. officinalis* barks (MOB), and evaluated different combinations of SBR and MOB for a reduction in cytotoxicity. The antioxidant, antimicrobial, and anti-inflammatory activities of the individual compounds and combinations thereof were also assessed.

## 2. Materials and Methods

### 2.1. Extract Preparation

Dried SBR and MOB were purchased from Phytoway Inc. (Hunan, China). The dried roots and barks were then separately ground using a laboratory rotor mill pulviresette (Laval Lab Inc., Laval, QC, Canada), and the pulverized products were sterilized by spraying them with 70% ethanol, followed by drying at 80 °C for 24 h and storage at 4 °C. All extract preparation steps were performed separately for SBR and MOB.

### 2.2. Extract Separation

To optimize the extraction of baicalin from SBR and magnolol and honokiol from MOB, different ethanol concentrations and time periods were tested (see Tables S1 and S2). In addition, SBR extraction with different concentrations of phosphoric acid for different time periods was tested. MOB was fractionated with different solvents (butanol, chloroform, acetone, and hexane) for different time periods. Extracts were then obtained using the optimal conditions, as described below.

Finely ground SBR powder was extracted with 0% ethanol at 80 °C for 2 h. The ratio of root powder to water was 1:8 (*w*/*v*). The resulting slurries were filtered through Whatman Grade 1 filter paper. This procedure was repeated twice for the residue and the filtrates were combined. The extracted solution was further treated with 1.5% phosphoric acid for 30 min. The solvent was removed under reduced pressure in a rotary evaporator (Rotavapor; BUCHI Labortechnik AG, Flawil, Switzerland) and freeze-dried (Labconco, Kansas City, MO, USA). The obtained extract powder was desiccated and stored at 4 °C and used directly in the antioxidant tests. Each fraction was dissolved in 10% dimethyl sulfoxide (DMSO) and highly concentrated stock solutions were prepared to ensure that the DMSO concentration did not exceed 0.1% in any of the experiments.

Finely ground MOB powder was extracted with 50% ethanol at 60 °C for 2 h. The ratio of bark powder to ethanol was 1:8 (*w*/*v*). Filtration steps were performed as described earlier for SBR extraction. The extracted solution was then treated with hexane for 30 min. The solvent was removed through evaporation and desiccated storage. Stock solution was prepared according to the methods explained earlier.

GenoTX-407 was prepared by combining SBR and MOB extracts in a 5:1 ratio (*w*/*w*) before further analysis.

### 2.3. High-Performance Liquid Chromatography (HPLC) Analysis

Each sample was weighed in a volumetric flask and 50 mL of 10% methanol was added. The solution was then sonicated for 60 min. After cooling, the solution was filtered using a 0.2 μm syringe filter (PVDF, Whatman plc, Maidstone, UK) and injected into the ACQUITY ultra-high-performance liquid chromatography (UHPLC) system (Waters Co., Milford, MA, USA), which was equipped with a binary solvent delivery pump, an autosampler, and a photodiode array detector. The output signal was monitored at a wavelength of 276 nm and processed using Empower 2 software (Waters Inc., Pleasanton, CA, USA). Chromatographic separation was performed using a Supelco Discovery C18 column (5 μm, 4.6 × 250 mm; Supleco, Taiwan, ROC). The column and autosampler tray temperatures were maintained at 25 °C. For SBR extract and GenoTX-407, the mobile phases A and B were 1% H_3_PO_4_ (*v*/*v*) and CH_3_CN, respectively. Gradient elution was performed as follows: 0–10 min with 5–50% solvent B, 10–20 min with 50–70% solvent B, 20–50 min with 70–100% solvent B. For MOB extract and GenoTX-407, the mobile phases A and B were Water and Methanol, respectively. Gradient elution was performed as follows: 0–10 min with 5–50% solvent B, 10–20 min with 50–70% solvent B, 20–50 min with 70–100% solvent B.

### 2.4. Cell Culture

RAW 264.7 (mouse leukemic monocyte macrophage) cell line and HeLa cells were cultured in Dulbecco’s modified Eagle’s medium (DMEM; Biowest, Nuaillé, France) containing antibiotics (100 IU/mL penicillin and 100 µg/mL streptomycin) and 10% heat-inactivated fetal bovine serum (Biowest, Nuaillé, France) at 37 °C under 5% CO_2_/95% air atmosphere. In all experiments, cells were grown until more than 90% confluent and subjected to no more than 20 passages.

### 2.5. 3-(4,5-Dimethylthiazol-2-yl)-2,5-Diphenyltetrazolium Bromide (MTT) Assay

The cytotoxicity of SBR, MOB, and GenoTX-407 was determined via the MTT assay. Briefly, RAW 264.7 cells (1 × 10^6^ cells/mL) were seeded into each well of a 96-well plate. After 24 h incubation at 37 °C in a 5% CO_2_/95% air atmosphere, the cells were treated with various concentrations (0–1000 µg/mL) of SRB and MOB extracts and GenoTX-407, then further incubated for 24 h. The cells were then washed twice with a fresh medium, followed by the addition of 10 μL of MTT (5 mg/mL in DMEM) and incubation for an additional 2 h. The medium was discarded, and the formazan formed in the living cells was dissolved in 50 μL DMSO. Absorbance was measured at 570 nm within 30 min using a microplate reader. The experiments were performed in triplicate and mean and standard deviation values were calculated.

### 2.6. Quantitative Real-Time Polymerase Chain Reaction (qRT-PCR)

Total RNA was isolated from cells and brain tissue using TRIzol reagent (Invitrogen, Carlsbad, CA, USA) according to the manufacturer’s instructions. qRT-PCR was performed according to previously described methods. Glyceraldehyde 3-phosphate dehydrogenase (GAPDH) was used as an internal standard to normalize the expression of the target transcripts. The primer sets used to amplify tumor necrosis factor-alpha (TNF-α), inducible nitric oxide synthase (iNOS), nuclear factor-κB (NF-κB), prostaglandin-endoperoxide synthase 2 (COX2), and interleukin-6 (IL-6) are shown in Table 1. Triplicate data were analyzed by five independent assays using the comparative Ct method.

### 2.7. Nitric Oxide Production in RAW 264.7 Cells

RAW264.7 cells (1 × 10^6^ cells/mL) were seeded in a 24-well tissue culture plate and pre-incubated at 37 °C for 12 h to achieve stable attachment. Next, the wells were washed with phosphate-buffered saline (PBS), refreshed with media containing lipopolysaccharide (LPS, 1 µg/mL), SBR (25, 50, and 100 µg/mL), MOB (5, 10, and 20 µg/mL), and GenoTX-407 (20 and 100 µg/mL), and incubated for 24 h. Nitric oxide (NO) production was then monitored by measuring nitrite levels in the culture media using the Griess Reagent System (Promega Corporation, Madison, WI, USA).

### 2.8. Test Microorganisms and Growth Conditions

The common pathogenic strains used to evaluate the antimicrobial effects of the extracts were gram-positive bacteria (*Staphylococcus aureus* ATCC 6538, *Staphylococcus epidermidis* ATCC 12228, *Bacillus subtilis* ATCC 33608, and *Propionibacterium acnes* ATCC 9027), gram-negative bacteria (*Escherichia coli* ATCC 8739, *Pseudomonas aeruginosa* ATCC 9097), yeasts (*Candida albicans* ATCC 10231, *Saccharomyces cerevisiae* ATCC 13007), and fungi (*Aspergillus niger* ATCC 16404, *Trichophyton rubrum* ATCC 62345) obtained either from the Korean Culture Center of Microorganisms (KCCM, Seoul, Korea) or the Korean Collection for Type Cultures (KCTC, Jeongeup-si, Korea). *Escherichia coli, B. subtilis*, and *P. aeruginosa* were cultured on nutrient agar (BD Difco, NJ, USA.); *S. aureus* and *S. epidermidis* were cultured on trypticase^TM^ soy agar (BD Difco, NJ, USA); and *S. epidermidis* was cultured on yeast mold (YM) agar (BD Difco, NJ, USA.) for 24 h at 37 °C after inoculation. *Candida albicans* and *T. rubrum* were cultured on YM agar and *A. niger* was cultured on potato dextrose agar BD Difco, NJ, USA) for 3 to 5 days at 30 °C after inoculation. *Propionibacterium acnes* was cultured on reinforced clostridial agar (Oxoid Ltd., Hampshire, UK) for 2 to 3 days at 25 °C after inoculation under anaerobic conditions in an anaerobic jar containing AnaeroGen sachets (Oxoid Ltd., Hampshire, UK) to maintain anaerobic conditions.

### 2.9. Agar Disk Diffusion Assay

Antimicrobial tests involved agar disk diffusion using 100 µL of a suspension containing 10^7^ CFU/mL bacteria and 10^6^ CFU/mL yeast, spread onto the respective agar plates [19]. The disks (8 mm in diameter, ADVANTEC CO., LTD., Tokyo, Japan) were impregnated with 20 µL of the 100 mg/mL extracts (2 mg/disk) and placed on the inoculated agar. DMSO-loaded disks were used as negative controls. The inoculated plates were incubated at 37 °C for 24 h in case of bacteria and at 30 °C for 2 to 5 days in case of yeast and fungi. Antibacterial activity was evaluated by measuring the diameter of the clear zones around the disks. Antibacterial activity was expressed as the mean zone of inhibition diameters (mm) produced by the extract. Each assay in this experiment was repeated three times.

### 2.10. Minimum Inhibitory Concentration (MIC) and Minimum Bactericidal Concentration (MBC) Determination

The minimum inhibitory concentration (MIC) values were determined for the microbial strains that were sensitive to the extracts in the agar disk diffusion assay. Test microbial strains were incubated using broth media. The absorbances of the cultured suspensions were measured at 660 nm using a spectrophotometer and diluted to attain viable cell counts of 10^6^ CFU/mL for bacteria and 10^5^ CFU/mL for yeast and fungi. The extracts were dissolved in DMSO, then diluted to the highest stock concentration (100 mg/mL), followed by serial two-fold dilutions to obtain stocks with concentrations ranging from 0.0031 to 50 mg/mL in 10 mL sterile test tubes containing DMSO. The MIC values of the extracts against bacterial strains or yeasts were determined using a previously described microwell dilution method with some modifications [20].

In 96-well plates, 185 µL of broth and 5 µL of the inocula were dispended into each well. Then, 10 µL of extract dissolved in DMSO (0.0031–100 mg/mL) was added into each well. The final volume in each well was 200 µL and the final concentrations of the extracts ranged from 0.153–5000 µg/mL. The contents of each well were thoroughly mixed using a multichannel pipette and the microplates were incubated at 37 °C for 24 h for bacterial strains and at 30 °C for 2 to 5 days for yeast and fungi. Microbial cell viability and MIC values were determined by observing the turbidity of the suspension post-incubation. The minimum bactericidal concentrations (MBC) of the extracts were determined by taking a loopful of inoculum from each well of the microtiter plates with clear contents and streaking it onto the specific agar plates for bacterial and yeast species. The bacteria and yeast-streaked plates were incubated under the same respective incubation conditions as described previously. Then, the lowest concentrations of the extracts that inhibited growth of bacteria or yeast were considered as the MBCs.

### 2.11. Measurement of Radical Scavenging Activity

The 1,1-diphenyl-2-picrylhydrazyl (DPPH) (Sigma-Aldrich, St. Louis, MO, USA) radical scavenging activity of pyrogallol was measured as described previously [21]. Specifically, various concentrations of SBR, MOB, and GenoTX-407 (0–1000 µg/mL) were dissolved in distilled water, and 10 µL from the resulting solutions was dispensed into separate wells in a 96-well plate, then 190 µL of 200 mM DPPH in methanol was added to the wells. The mixtures were incubated at room temperature for 30 min and the absorbances of the reaction mixtures were measured at 517 nm with a microplate reader (Molecular Devices, San Jose, CA, USA). DPPH radical scavenging activity was expressed using the following formula: % scavenging activity = (control absorbance—sample absorbance)/control absorbance × 100. Ascorbic acid (Sigma-Aldrich) was used as a reference control. All samples were analyzed in triplicate.

### 2.12. Measurement of Lipid Peroxidation

Lipid peroxidation was measured by determining the formation of thiobarbituric acid-reactive substances (TBARS) in the brain. Briefly, the brains of ICR mice were dissected immediately after intracardial perfusion with cold phosphate-buffered saline (PBS). Brain tissue was homogenized in 10 volumes of cold PBS and centrifuged at 3000 rpm for 20 min at 4 °C to obtain the supernatant. To induce lipid peroxidation, 450 µL of the brain homogenate was mixed with 25 µL 50 µM ferric chloride in the presence or absence of various concentrations of SBR, MOB, and GenoTX-407 (25 µL) and incubated for 30 min at 37 °C. Sodium dodecyl sulfate (500 μL of an 8.1% *w*/*v* solution) and 1 mL of 20% acetic acid (pH 3.5) were added to the brain homogenate and centrifuged. Aliquots of the clear supernatant were mixed with equal volumes of thiobarbituric acid solution (0.8% *w*/*v*) and heated in a glass tube capped with aluminum foil at 95 °C for 30 min. Samples were cooled on ice, then 100 μL of each sample was pipetted into 96-well plates, and the absorbance was measured at 532 nm with a microplate reader.

### 2.13. Western Blot Analysis

HeLa cells were seeded in a 6-well tissue culture plate and pre-incubated at 37 °C for 12 h to achieve stable attachment. Next, the wells were washed with PBS, refreshed with media containing either SBR (100 µg/mL) or MOB (20 µg/mL) or GenoTX-407 (20, 50 and 100 µg/mL), and incubated for 24 h. HeLa cells were homogenized in 10 volumes of radioimmunoprecipitation assay (RIPA) buffer (Sigma-Aldrich) containing protease inhibitors (Sigma-Aldrich) and phosphatase inhibitors (Sigma-Aldrich). Western blot analysis was conducted as previously described [5]. The membranes were then immunoblotted with primary antibodies, followed by incubation with horseradish peroxidase-conjugated anti-rabbit antibodies (Cell signaling Technology, Danvers, MA, USA). The antibodies used in this study are Nrf2 (Abcam, Cambridge, UK), HO-1 (Abcam), SOD1 (Abcam), and CAT (Abcam). The band densities were measured using ImageJ software (National Institutes of Health, NIH, MD, USA) and normalized to the density of actin.

### 2.14. Statistical Analysis

Statistical comparisons between the groups were performed using one-way analysis of variance (ANOVA) followed by the Tukey’s multiple comparison test. All analyses were conducted using the Statistical Package for Social Sciences software, version 12.0 (SPSS Inc., Chicago, IL, USA). Statistical significance was recognized for *p* < 0.05. All data are expressed as mean ± standard deviation (SD).

## 3. Results

### 3.1. Preparation and Separation of Extracts

**Figure 1:**
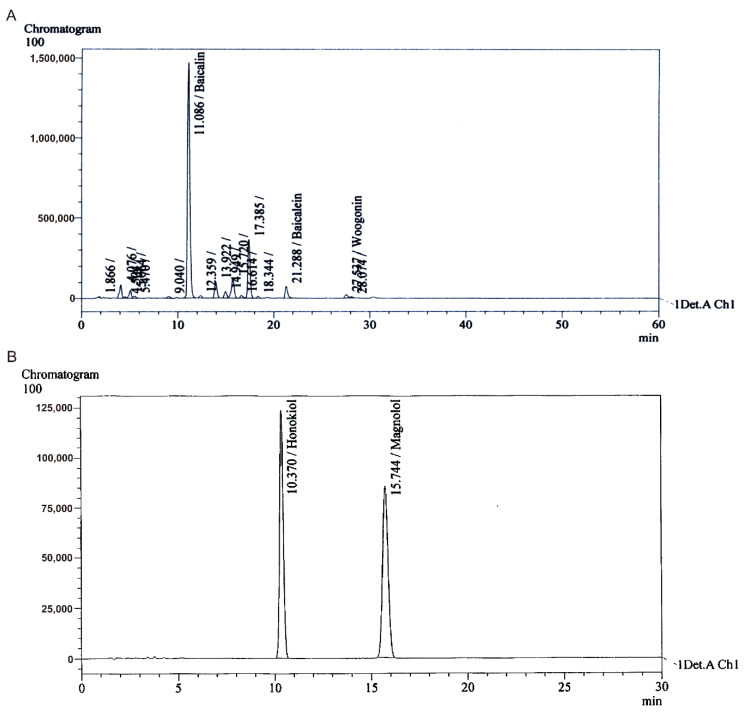
High-performance liquid chromatography of GenoTX-407. Peaks indicate baicalin existing in high proportion (**A**) and honokiol and magnolol existing in high proportion (**B**) in GenoTX-407.

To optimize the extraction of phenolic compounds from SBR and MOB, various ethanol concentrations and extraction times (see Tables S1 and S2), and treatments with different solvents (butanol, chloroform, acetone, and hexane) for different time periods were tested. The total baicalin content (64.2%) in SBR extract was maximized via extraction with 0% ethanol for 2 h followed by treatment with 1.5% phosphoric acid for 0.5 h. Extraction with 50% ethanol for 2 h followed by hexane treatment for 0.5 h resulted in the MOB extract with the highest total magnolol and honokiol contents (51.2%). Each polyphenol in the extracts of GenoTX-407 (Figure 1), SBR, and MOB (Figure S1) was identified by HPLC.

### 3.2. Anti-Inflammatory Effects

**Figure 2:**
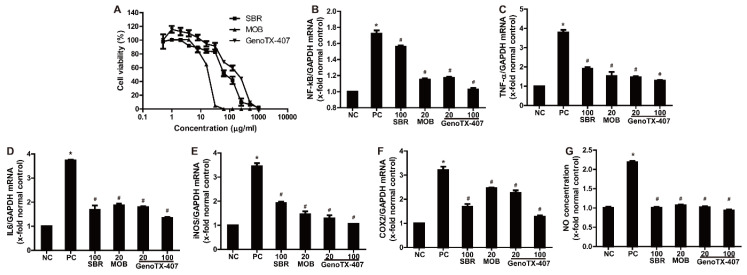
Anti-inflammatory activities of SBR, MOB, and GenoTX-407. (**A**) Cell viability in response to SBR, MOB, and GenoTX407 in the MTT assay. (**B**–**F**) Real-time PCR for checking the mRNA expression of *NF-κB* (**B**), *TNF-α* (**C**), *IL-6* (**D**), *iNOS* (**E**), and *COX2* (**F**), normalized to *GAPDH*. (**G**) Inhibitory effect on LPS-induced NO generation. *Significantly different from normal controls (*p* < 0.05). # Significantly different from positive controls (*p* < 0.05). SBR, *Scutellaria baicalensis* root; MOB, *Magnolia officinalis* barks; GenoTX-407, combination of SBR and MOB; LPS, lipopolysaccharide.

To evaluate cytotoxicity, the viability of RAW 264.7 cells was determined by an MTT assay after treatment with various concentrations (0–1000 μg/mL) of SBR and MOB extracts. As shown in Figure 2A, low concentrations (0–10 μg/mL) did not significantly influence cell viability. The concentrations required to inhibit cell viability by 50% were 95.46 and 15.89 μg/mL of SBR and MOB, respectively. At SBR and MOB concentrations of 250 and 31.3 μg/mL, respectively, RAW 264.7 growth was almost completely inhibited. The LD_50_ of GenoTX-407 was 131.9 μg/mL. Based on these data, we analyzed the anti-inflammatory and antioxidative effects of SBR (25, 50, and 100 μg/mL), MOB (5, 10, and 20 μg/mL), and GenoTX-407 (20 and 100 μg/mL).

To analyze the anti-inflammatory effects of SBR, MOB, and GenoTX-407, we investigated their effects on the NF-κB pathway in RAW264.7 cells by qRT-PCR. NF-κB expression was significantly higher in LPS-treated cells than that in the non-treated controls (Figure 2). NF-κB levels were dose-dependently reduced in cells co-treated with SBR or MOB and LPS compared to those in cells treated with LPS alone (Figures S2 and S3). Interestingly, treatment with GenoTX-407 showed the highest inhibitory effect on LPS-induced NF-κB upregulation. In parallel with the change in NF-κB levels, the expression of TNF-α and IL-6 was also remarkably increased by LPS; treatment with SBR, MOB, or GenoTX-407 blocked this LPS-mediated increase in TNF- α and IL-6 levels. Considering the upregulation of NF-κB and pro-inflammatory cytokines, gene expression levels of the inflammatory enzymes iNOS and COX2 were increased following exposure to LPS. Notably, treatment with SBR, MOB, and GenoTX-407 significantly inhibited the expression of these enzymes in a concentration-dependent manner. Similarly, LPS treatment remarkably upregulated NO levels, and co-treatment with SBR, MOB, and GenoTX-407 inhibited this upregulation.

### 3.3. Antimicrobial Activity

Agar disk diffusion and microdilution methods were used for determining the antimicrobial activities of SBR, MOB, and GenoTX-407 against pathogenic microorganisms; the results are summarized in Table 2 and Table 3. The obtained results revealed a sensitivity variation among the microorganisms tested (Figure S4). Particularly, GenoTX-407 showed the highest antimicrobial activity against all bacteria and fungi used in this study. In the disk diffusion assay, SBR did not exhibit antimicrobial activity against any of the microorganisms, although a MIC of 2500 μg/mL was recorded against *E. coli* in the microdilution assay. In general, MOB was less effective than GenoTX-407 and had no influence on the growth of the gram-negative bacteria, *E. coli* and *P. aeruginosa*. However, only GenoTX-407 showed antimicrobial activity against *E. coli* (10.20 ± 0.131 mm) and *P. aeruginosa* (9.89 ± 0.083 mm). The MBC values further revealed that GenoTX-407 exhibited bactericidal effects. Among the gram-positive bacteria, *P. acnes* was the most sensitive strain to MOB and GenoTX-407 treatment, exhibiting inhibition zones of 30.54 ± 0.922 and 35.10 ± 0.676 mm, respectively. The MBC values indicated that MOB and GenoTX-407 exhibited bacteriostatic effects against *P. acnes*, while *S. aureus* was the most resistant, with inhibition zones of 16.53 ± 0.361 and 18.90 ± 0.335 mm, respectively. Furthermore, *S. epidermidis* and *B. subtilis* were less sensitive to MOB and GenoTX-407 treatment.

MOB and GenoTX-407 exhibited notable activity against the yeasts *C. albicans* (16.48 ± 0.055 and 19.18 ± 0.155 mm) and *S. cerevisiae* (22.61 ± 1.266 and 28.12 ± 0.829 mm), respectively. MOB and GenoTX-407 also exhibited activities against *A. niger* (17.03 ± 0.871 and 18.92 ± 0.472 mm) and *T. rubrum* (49.77 ± 0.240 and 50.47 ± 0.200 mm), respectively.

### 3.4. Antioxidant Activity

**Figure 3:**
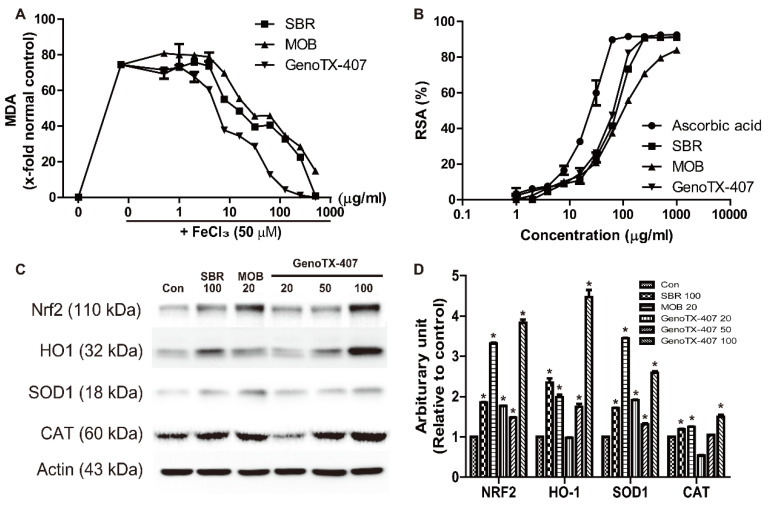
Antioxidant activities of SBR, MOB, and GenoTX-407. (**A**) Inhibitory effect on FeCl_3_-induced lipid peroxidation (TBARS production) (**B**) 1,1-diphenyl-2-picrylhydrazyl (DPPH) radical-scavenging activity in 30 min. (**C**,**D**) Western blotting to check the expression of Nrf2, HO-1, SOD1, and CAT in response to individual treatments (**C**). Band densities normalized to GAPDH (**D**). * Significantly different from vehicle controls (*p* < 0.05). SBR, *Scutellaria baicalensis* root; MOB, *Magnolia officinalis* barks; GenoTX-407, combination of SBR and MOB.

The antioxidant activities of SBR, MOB, and GenoTX-407 were examined using lipid peroxidation and 1,1-diphenyl-2-picrylhydrazyl (DPPH) assays. As shown in Figure 3, an increase (74%) in TBARS levels was observed after exposure to FeCl_3_ (50 µM). However, treatment with various concentrations (0–500 μg/mL) of SBR, MOB, and GenoTX-406 significantly reduced lipid peroxidation in a concentration-dependent manner. Similarly, in the DPPH assay, the treatments significantly increased radical scavenging activity in a dose-dependent manner. Interestingly, the results showed that GenoTX-407 had a higher capacity to reduce lipid peroxidation and DPPH (EC_50_, 7.7 µg/mL and 26.4 µg/mL) than SBR (107.7 µg/mL and 38.3 µg/mL), and MOB (62.4 µg/mL and 72.5 µg/mL). However, in the DPPH assay, all the treatments were less potent than the treatment agent used in positive control, ascorbic acid (14.4 µg/mL). In the western blot analysis, the expression of the antioxidative enzymes SOD1 and CAT, as well as Nrf2 and HO-1, was markedly upregulated in response to treatments with SBR, MOB, and GenoTX-407. Notably, GenoTX-407 treatment (100 µg/mL) resulted in higher upregulation of these enzymes compared to SBR (100 µg/mL) and MOB (20 µg/mL).

## 4. Discussion

In this study, we demonstrated that SBR, MOB, and GenoTX-407 exhibited antioxidant, antimicrobial, and anti-inflammatory effects in a concentration-dependent manner. Interestingly, SBR exhibited more potent antioxidant activity than MOB. Contrastingly, MOB exhibited more potent anti-inflammatory and antimicrobial activities than SBR. In the antioxidant and anti-inflammatory tests, both SBR and MOB showed concentration-dependent effects, and the proportion was determined at 5:1 (SBR: MOB) based on the cytotoxicity test. GenoTX-407, a combination of SBR and MOB extracts, revealed a synergistic effect of SBR and MOB in the antioxidant and anti-inflammatory activity tests. Moreover, in comparison to MOB, GenoTX-407 showed higher antimicrobial activity against all bacteria and fungi investigated in this study.

It is well known that baicalin is the predominant flavonoid in SBR and it has a defined chemical structure [22]. In this study, we tried to determine the optimal conditions for extraction of baicalin from SBR and MOB. Although several factors influence baicalin extraction, previous studies have revealed that extraction time is the major determining factor in optimizing the extraction process [23,24]. Therefore, in our study, the effects of two experimental factors, i.e., ethanol concentration and extraction time, on baicalin yield from SBR were studied using HPLC. Then, we verified the stability and precision of the optimized extraction process by determining the optimum conditions, including ethanol concentration (0%) and extraction time (2 h). Magnolol and honokiol are major components isolated from MOB and the optimized conditions for their extraction are 50% ethanol concentration and 2 h extraction time [25].

Baicalin is a major component in SBR and exhibits various pharmacological activities, including antioxidative, antiviral, anti-inflammatory, and antiproliferative effects [26,27,28]. In this study, SBR also exhibited potent antioxidant effects, as determined in the DPPH assay and lipid peroxidation assay. It is thought that the antioxidant effects of SBR are mediated by Nrf2/HO-1 pathway and upregulation of antioxidant enzymes. It is well-known that Nrf2 is a key regulator of oxidative stress and baicalin ameliorates oxidative stress and apoptosis during endothelial impairment, liver injury, and brain injury by activating the Nrf2/HO-1 antioxidative pathway [9,29,30,31]. Moreover, it has been reported that honokiol and magnolol in MOB exhibit potent antioxidative, anti-inflammatory, antitumor, and antimicrobial effects via several modes of action [25,32,33,34]. In particular, their anti-inflammatory effects are mediated via the inhibition of the NF-κB signaling pathway [13]. Furthermore, we confirmed that the expression of key NF-κB signaling pathway intermediaries, i.e., COX2, IL6, and TNF-α was decreased in this study. Interestingly, GenoTX-407 exhibited the most potent antioxidant and anti-inflammatory effects. Apparently, the synergic effect of GenoTX-407 is the result of Nrf2 upregulation by SBR extract and inhibition of NF-κB signaling pathway by MOB extract. It has been reported that the activation of Nrf2 attenuates the NF-κB-induced inflammatory response [35]. In addition, GenoTX-407 exhibited lower cytotoxicity than SBR and MOB, likely due to a relatively low dose.

Moreover, treatment with SBR was less effective against bacteria, compared to MOB treatment. Although MOB exhibited activity against some bacteria, GenoTX-407 exhibited the highest activity against all bacteria and fungi that were investigated in this study. In many studies, co-treatment with baicalin enhanced the growth-inhibitory effects of antibiotics [36]. Baicalin interferes with cell wall integrity through direct binding to peptidoglycans and exerts synergistic antimicrobial activity with tetracycline and β-lactams [36,37]. Besides, it has been reported that baicalin restores the inhibition of TetK-mediated tetracycline efflux [36,37]. Similarly, magnolol and honokiol in MOB extract have been known to show high antimicrobial activity against several microorganisms, such as fungi, *Propionibacterium* species, and *S. aureus* [12,38]. SBR and MOB exhibit synergistic effects with several antibiotics, such as oxacillin, ampicillin, chloramphenicol, tetracycline, and cefoxitin [39]. Thus, the combination of SBR and MOB (GenoTX-407) had the most potent antimicrobial activity in this study.

## 5. Conclusions

Taken together, the results obtained in this study suggest that GenoTX-407, the combination of SBR and MOB, is a potential drug candidate exerting antioxidant and anti-inflammatory effects via the Nrf2/HO-1 and NF-κB signaling pathways and insights gleaned from these studies could lead to the development of novel combination drug therapies for the treatment of various diseases

## Figures and Tables

**Table 1:** Sequences of the primers used in the current study.

		Mouse Primer	
Gene Name	Accession No.	Forward (5′–3′)	Reverse (5′–3′)
*COX2*	NM_011198	GAACCTGCAGTTTGCTGTGG	ACTCTGTTGTGCTCCCGAAG
*IL-6*	NM_031168.1	TCCAGTTGCCTTCTTGGGAC	AGTCTCCTCTCCGGACTTGT
*iNOS*	NM_010927.3	CTATGGCCGCTTTGATGTGC	TTGGGATGCTCCATGGTCAC
*NF-κB*	NM_008689	CACTGCTCAGGTCCACTGTC	CTGTCACTATCCCGGAGTTCA
*TNFα*	NM_013693	TACCTTGTTGCCTCCTCTT	GTCACCAAATCAGCGTTATTAAG
*GAPDH*	NM_008084	CGTGCCGCCTGGAGAAACC	TGGAAGAGTGGGAGTTGCTGTTG

**Table 2:** Antimicrobial activities of extracts of *Scutellaria baicalensis* root (SBR), *Magnolia officinalis* bark (MOB), and the combination of SBR and MOB, i.e., GenoTX-407, determined using the agar disk diffusion assay.

Inhibition Zones Diameter (mm) * around Disks Impregnated with 20 μL of Each Extract (2 mg/disk)				
Microbial Strains	DMSO	SBR	MOB	GenoTX-407
*Escherichia coli* ATCC 8739	ND	ND	ND	10.20 ± 0.131
*Staphylococcus aureus* ATCC 6538	ND	ND	16.53 ± 0.361	18.90 ± 0.335
*Candida albicans* ATCC 10231	ND	ND	16.48 ± 0.055	19.18 ± 0.155
*Propionibacterium acnes* ATCC 9027	ND	ND	30.54 ± 0.922	35.10 ± 0.676
*Staphylococcus epidermidis* ATCC 12228	ND	ND	19.10 ± 0.259	24.04 ± 0.492
*Pseudomonas aeruginosa* ATCC 9097	ND	ND	ND	9.89 ± 0.083
*Bacillus subtilis* ATCC 33608	ND	ND	20.70 ± 0.516	24.41 ± 0.492
*Saccharomyces cerevisiae* ATCC 13007	ND	ND	22.61 ± 1.266	28.12 ± 0.829
*Aspergillus niger* ATCC 16404	ND	ND	17.03 ± 0.871	18.92 ± 0.472
*T. rubum* ATCC 62345	ND	ND	49.77 ± 0.240	50.47 ± 0.200

* The diameter of the inhibition zones (mm), including the diameter of the disks (8 mm), are presented as mean ± SD of triplicate experiments; DMSO: dimethyl sulfoxide used as control; ND; not detected.

**Table 3:** The Minimum inhibitory concentration (MIC) and minimum bactericidal concentration (MBC) (μg/mL) of SBR, MOB, and GenoTX-407, a combination of SBR and MOB, against pathogenic microorganisms.

Microbial Strains	SBR		MOB		GenoTX-407	
	MIC	MBC	MIC	MBC	MIC	MBC
*Escherichia coli* ATCC 8739	2500	>5000	78.1	78.1	39.1	39.1
*Staphylococcus aureus* ATCC 6538	- *	NA	39.1	39.1	1.5	19.5
*Candida albicans* ATCC 10231	-	NA	9.77	9.77	2.44	2.44
*Propionibacterium acnes* ATCC 9027	-	NA	2.44	9.77	2.44	9.77
*Staphylococcus epidermidis* ATCC 12228	-	NA	4.88	4.88	4.88	4.88
*Pseudomonas aeruginosa* ATCC 9097	-	NA	-	NA	2500	2500
*Bacillus subtilis* ATCC 33608	-	NA	9.77	19.5	9.77	19.5
*Saccharomyces cerevisiae* ATCC 13007	-	NA	9.77	19.5	9.77	19.5
*Aspergillus niger* ATCC 16404	-	NA	78.1	78.1	78.1	78.1
*T. rubum* ATCC 62345	-	NA	4.88	4.88	4.88	4.88

* Not inhibited at concentration 5000 μg/mL. NA; not applicable.

## Supplementary Materials

The following are available online at https://www.mdpi.com/2076-3921/9/11/1111/s1, Table S1. The weights of extracts of SBR depending on different ethanol concentrations and time periods. Table S2. The weights of extracts of MOB depending on different ethanol concentrations and time periods. Figure S1 High-performance liquid chromatography of SBR (A) and MOB (B). Figure S2 Anti-inflammatory activity of SBR (25, 50, and 100 μg/mL). Figure S3 Anti-inflammatory activity of MOB (5, 10, and 20 μg/mL). Figure S4. Antimicrobial activity of SBR, MOB, and GenoTX-407.
