# Supplementary material for: Pharmaceutical Advantages of GenoTX-407, A Combination of Extracts from Scutellaria baicalensis Root and Magnolia officinalis Bark
Source: Antioxidants (Basel). 2020 Nov 11;9(11):1111. doi: 10.3390/antiox9111111 (PMC7697866; doi:10.3390/antiox9111111)
Supplement: Supplementary file 1 [file antioxidants-09-01111-s001.pdf]

Table S1. The weights of SBR extracts depending on different ethanol concentrations and time periods.

Ethanol	0%	20%	40%	50%	60%
1 h	13.1%	11.3%	10.3%	10.6%	10.2%
2 h	18.2%	13.2%	10.5%	10.3%	10.3%
3 h	18.0%	13.3%	9.4%	10.5%	10.2%
4 h	17.3%	13.6%	9.5%	10.2%	10.7%

The data indicate the ratio of relative weights to initial weights of SBR.

Table S2. The weights of MOB extracts depending on different ethanol concentrations and time periods.

Ethanol	0%	50%	70%	80%
1 h	12.0%	12.3%	11.2%	9.1%
2 h	12.2%	13.4%	11.0%	9.2%
3 h	12.1%	13.2%	11.3%	9.5%
4 h	12.2%	13.1%	11.3%	9.3%

The data indicate the ratio of relative weights to initial weights of MOB.

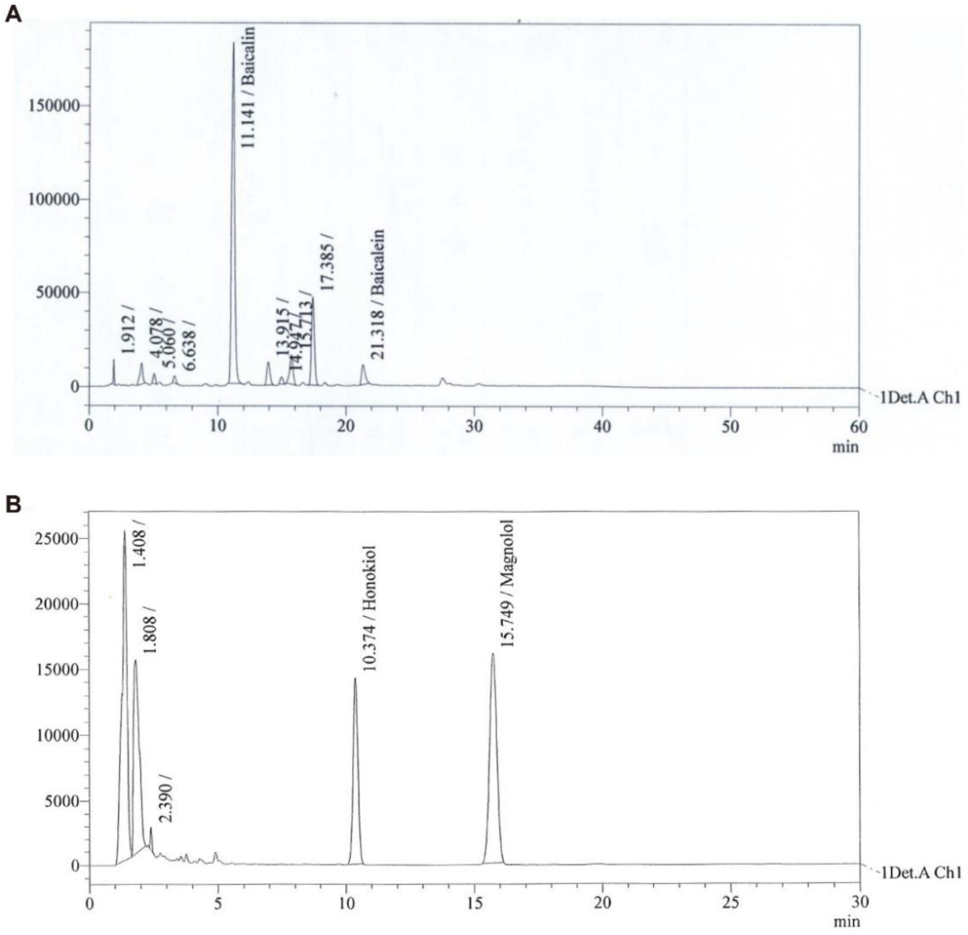

Figure S1. High-performance liquid chromatography of SBR (A) and MOB (B). Peaks indicate abundant baicalin in SBR and honokiol and magnolol in MOB. SBR, *Scutellaria baicalensis* root; MOB, *Magnolia officinalis* barks.

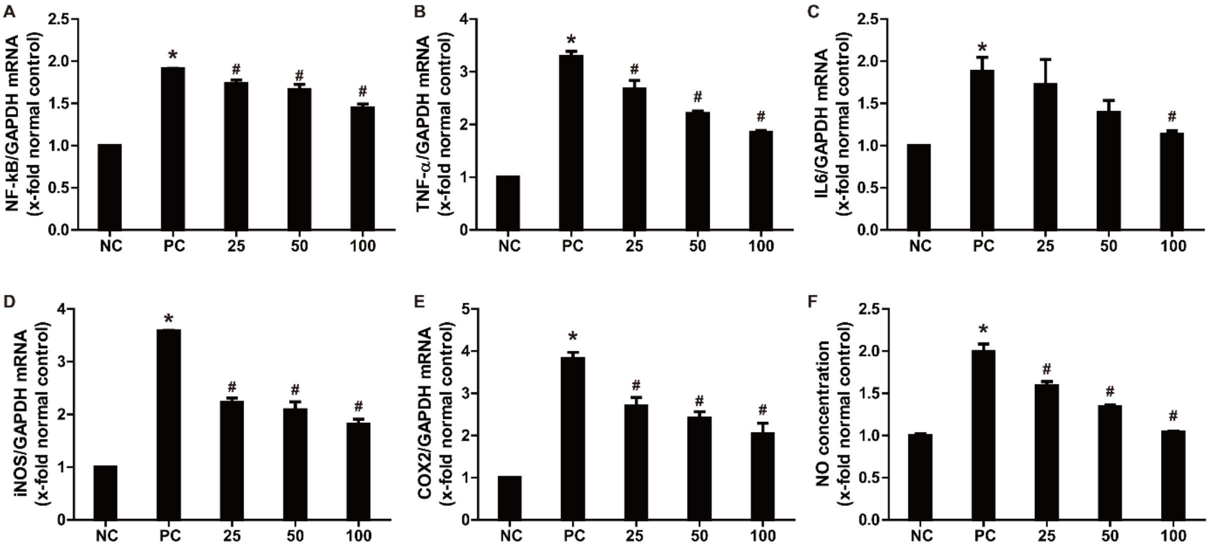

Figure S2. Anti-inflammatory activity of SBR (25, 50, and 100 μg/mL). Real-time PCR for checking the expression of *NF-κB* (A), *TNF-α* (B), *IL-6* (C), *iNOS* (D), and *COX2* (E) mRNA normalized against *GAPDH*, (F) Inhibitory effect on LPS-induced NO generation. *Significantly different from normal controls (P < 0.05). #Significantly different from positive controls (P < 0.05). SBR, *Scutellaria baicalensis* root; LPS, lipopolysaccharide

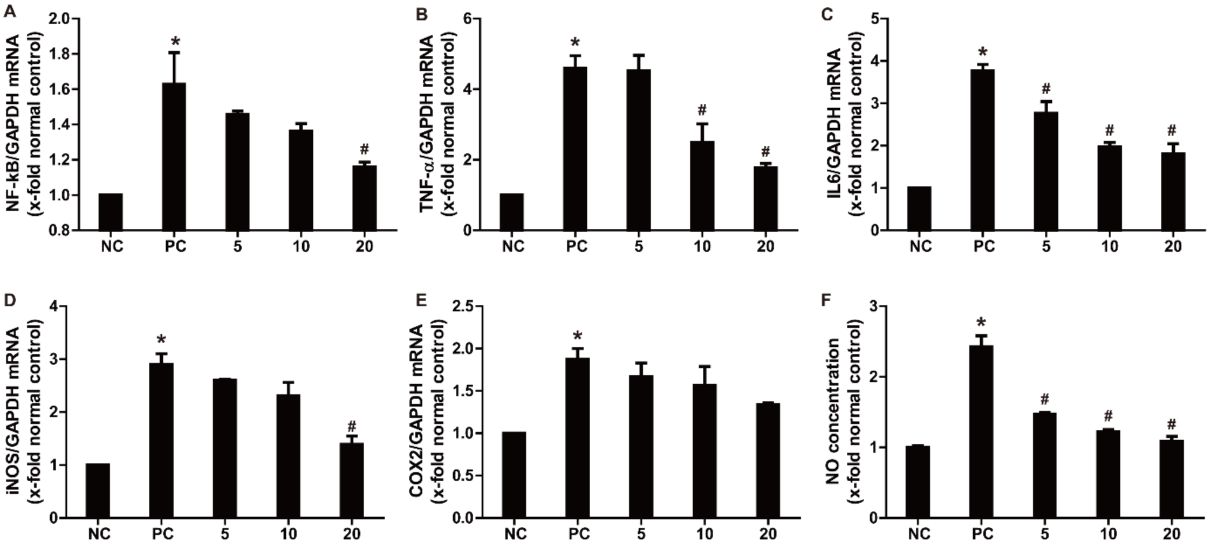

Figure S3. Anti-inflammatory activity of MOB (5, 10, and 20 μg/mL). Real-time PCR for checking the expression of *NF-κB* (A), *TNF-α* (B), *IL-6* (C), *iNOS* (D), and *COX2* (E) mRNA normalized against *GAPDH*. (F) Inhibitory effect on LPS-induced NO generation. *Significantly different from normal controls (P < 0.05). #Significantly different from positive controls (P < 0.05). MOB, *Magnolia officinalis* barks; LPS, lipopolysaccharide

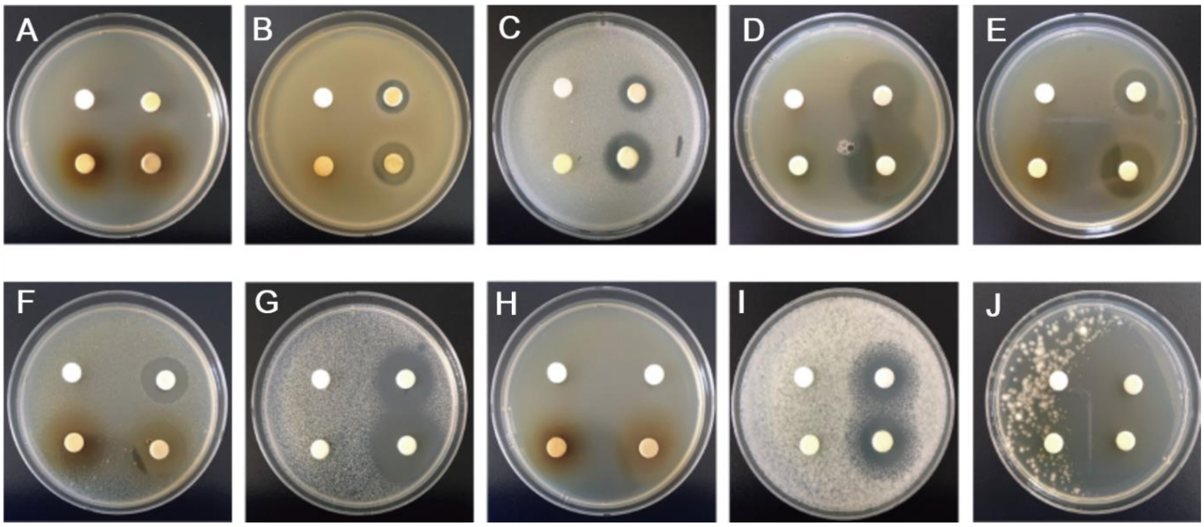

Figure S4. Antimicrobial activities of SBR, MOB, and GenoTX-407 against (A) *Escherichia coli*, (B) *Staphylococcus aureus*, (C) *Candida albicans*, (D) *Propionibacterium acnes*, (E) *Staphylococcus epidermidis*, (F) *Pseudomonas aeruginosa*, (G) *Bacillus subtilis*, (H) *Saccharomyces cerevisiae*, (I) *Aspergillus niger*, and (J) *T. rubum* determined using the agar disk diffusion method. Top: Left, DMSO; Right, SBR; Bottom: Left, MOB; Right, GenoTX-407. SBR, *Scutellaria baicalensis* root; MOB, *Magnolia officinalis* barks; GenoTX-407, combination of SBR and MOB
